# Climatic droplet keratopathy in both eyes: A case report

**DOI:** 10.1097/MD.0000000000043086

**Published:** 2025-07-11

**Authors:** A bu du sa ta er Ai shan, Dai Kun Lei, Ying Zhou, Xia Li

**Affiliations:** a Ophthalmic Centre, Xinjiang 474 Hospital, Urumqi, Xinjiang, China.

**Keywords:** case report, climatic droplet keratopathy, corneal decompensation, dystrophia epithelialis corneae

## Abstract

**Rationale:**

Climatic droplet keratopathy is characterized by the formation of tiny, oil-like droplets on the cornea’s surface, leading to discomfort and visual disturbances. This case report describes complex corneal opacity in the central pupillary zone, highlighting diagnostic challenges and the importance of early intervention.

**Patient concerns:**

The patient presented with persistent discomfort and progressive visual disturbances due to bilateral corneal opacity and epithelial changes.

**Diagnoses:**

Initial clinical manifestations raised suspicion for corneal decompensation. Definitive exclusion of endothelial dystrophy was achieved through diagnostic surgical intervention (epithelial scraping in the right eye), which demonstrated intact endothelial function via rapid postoperative healing. The final diagnosis include bilateral climatic droplet keratopathy with cataracts.

**Interventions:**

Elective staged surgical excision was performed, beginning with epithelial scraping combined with cataract surgery in the right eye, followed by strategic excision of the Bowman layer and superficial stroma within a 5 mm × 5 mm pupillary zone in both eyes, demonstrating the value of selective intervention.

**Outcomes:**

The surgical intervention achieved successful corneal re-epithelialization accompanied by significant visual acuity recovery. Serial follow-up over 12 months confirmed sustained restoration of central corneal transparency and stability in visual function.

**Lessons:**

This case establishes that diagnostic surgical scraping is essential to exclude endothelial dysfunction in climatic droplet keratopathy. Guided by this confirmation, targeted anterior lamellar keratectomy achieved successful visual rehabilitation, with sustained central corneal transparency objectively documented at 1-year follow-up.

## 1. Introduction

Climatic droplet keratopathy (CDK) is a degenerative ocular disease that primarily affects the cornea. Its development is strongly linked to environmental factors, rendering it a potentially blinding condition. The characteristic manifestation of CDK is corneal opacity, which arises from an abnormal accumulation of oil-like deposits on both the Bowman layer and stroma.^[1]^ The early stages of CDK often exhibit subtle clinical manifestations, which can be masked by the onset of more pronounced symptoms later in the disease course. In advanced stages, CDK typically presents as a deposition of opaque material that forms band-like structures covering the cornea, significantly impairing vision and posing a risk of blindness.^[2]^ There is no established pharmacological therapy for CDK. In terms of surgical interventions, superficial keratectomy in combination with amniotic membrane or corneal transplantation remains the primary treatment option for patients suffering from severe CDK.^[3]^ The disease can be classified into 3 stages based on corneal involvement and clinical features: Grade 1 is characterized by small, translucent deposits near the limbus, which are best visualized under backscattered slit-illumination. Notably, visual acuity remains unaffected in this stage. Grade 2: In this stage, a fuzzy opacity covers the lower two-thirds of the cornea, indicative of chronic exposure to ultraviolet radiation and stress. The superior cornea remains unaffected. A decline in visual acuity marks this stage. Grade 3: The most advanced stage of CDK is characterized by golden droplets covering the entire cornea, leading to opacification, vascularization, or fibrosis in severe cases. Visual acuity is severely impaired at this stage.^[4]^ A case of CDK in stage 2 is presented, highlighting its clinical features and treatment approach.

## 2. Case presentation

**Figure 1.**
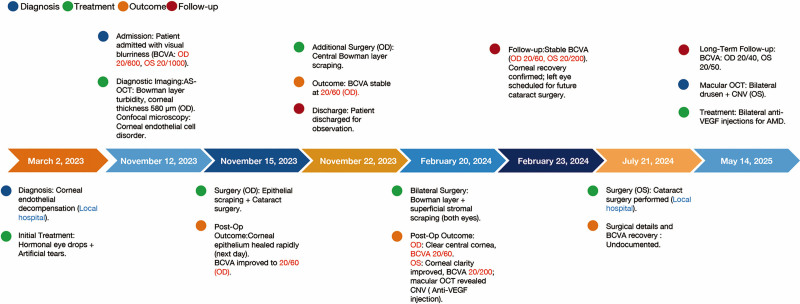
Timeline of events.

A 69-year-old male patient from the Kazakh ethnic group residing in the Altay region of Xinjiang, China, was admitted to the hospital on November 12, 2023, presenting with primary symptoms of visual blurriness. A young doctor consulted regarding the possibility of performing a corneal transplant, while the patient requested cataract surgery due to worsening blurred vision over the past 2 years. The patient reported no symptoms of pain or other precipitating factors and had previously been diagnosed with decompensation of the corneal endothelium at a local hospital. Despite receiving treatment with corticosteroid eye drops and artificial tears, the patient’s visual acuity continued to decline, ultimately leading to a steady deterioration. Consequently, the patient was referred to our institution for specialized treatment. The timeline of the patient care was shown in Figure 1.

**Figure 2.**
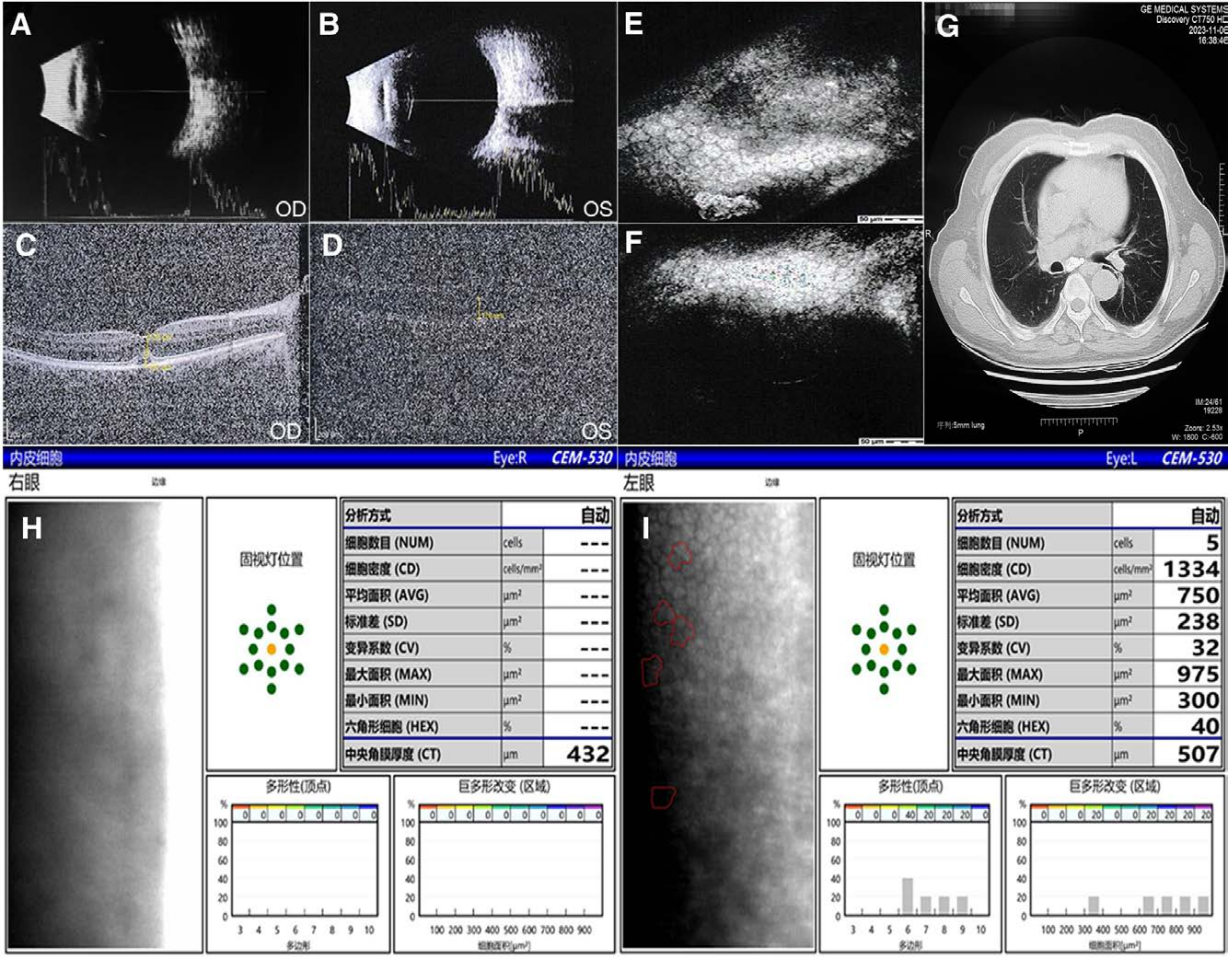
(A and B) Ocular ultrasonography showed normal optical axes, vitreous clouding in both eyes, bilateral cataracts. (C and D) Macular OCT showed detachment of the pigment epithelium in the macular area of the right eye, and neovascularization in the macular area was faintly visible in the left eye. (E and F) Corneal polyconfocal endothelial microscopic examination suggested hexagonal endothelial cells visible in both eyes. (G) Axial chest CT image demonstrating unremarkable pulmonary parenchyma without evidence of active inflammatory changes or structural abnormalities. (H and I) Corneal endothelial microscopy showed no endothelial cells in the right eye and a cell density of 750 cells/mm^2^ in the left eye.

**Figure 3.**
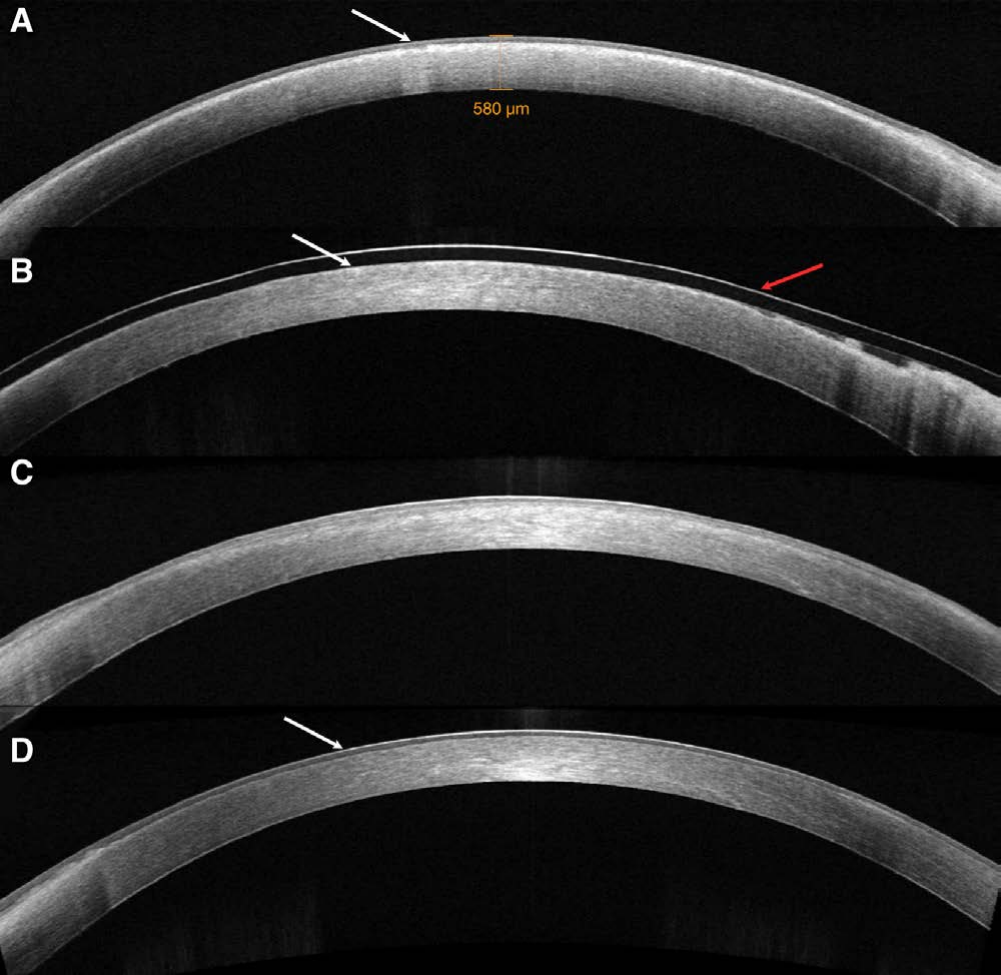
Anterior segment optical coherence tomography. (A) A preoperative clouding of the anterior elastic lamina with increased density (shown by white arrows). (B) Postoperative marked reduction of the clouding of the anterior elastic lamina (indicated by white arrows) and wearing a bandage contact lens (shown by red arrows). (C) Anterior elastic lamina clouding was significantly reduced at 1 month postoperatively. (D) At 1-year postoperative follow-up revealed complete restoration of the corneal epithelial layer with intact Bowman membrane architecture (white arrows).“

Physical examination revealed that the best-corrected visual acuity was 20/600 on the right eye and 20/1000 on the left eye. Mild conjunctival congestion was observed in both eyes. The lower two-thirds of the cornea were cloudy, with oil droplets intruding into the pupil area; the upper third remained clear in both eyes. The anterior chamber was transparent, and the pupil was round with a positive light reflex, approximately 3 mm in diameter. Both eyes exhibited yellowish-brown cataracts, and the intraocular structures were not visible. Ultrasonography revealed normal optical axes, clouding of the vitreous in both eyes, and bilateral cataracts (Fig. 2A and B). Anterior segment optical coherence tomography (AS-OCT) of the right eye revealed turbidity of the Bowman layer, and the central corneal thickness measured 580 µm (Fig. 3A). No valid data could be acquired from the corneal endothelium in the left eye; 750 corneal endothelial cells were counted by the corneal endothelial scope of the right eye (Fig. 2H and I). Confocal microscopic cornea examination revealed corneal endothelial cell disorder (Fig. 2E and F). The rest of the general examination and test results were not abnormal. According to the diagnosis of corneal decompensation and treatment in the local hospital, the conditions of the eye's anterior segment, and the auxiliary examination results, our initial considerations for diagnosis were as follows: Dystrophia epithelial cornea in both eyes; Cataract in both eyes. The patient initially presented with visual blur, and imaging results revealed phacoscotasmus; these observations were in line with the diagnostic criteria for Cataract. AS-OCT showed turbidity of the Bowman layer, and normal thickness of 580 µm. So, the final diagnosis of the presented case is as follows: CDK in both eyes; Cataract in both eyes. In histopathological light microscope examination, globular deposits of different sizes may be observed under the corneal epithelium, within the Bowman membrane, and in the anterior stroma. The coalescence and increased volume of these spherules or deposits may disrupt the Bowman membrane, and elevation and thinning of the corneal epithelium. Limited chest CT was obtained per patient preference to evaluate post-influenza status, demonstrating normal pulmonary parenchyma (Fig. 1G). We also performed eye macular optical coherence tomography (macular OCT), AS-OCT, and optometry, and measured the intraocular pressure.

**Figure 4.**
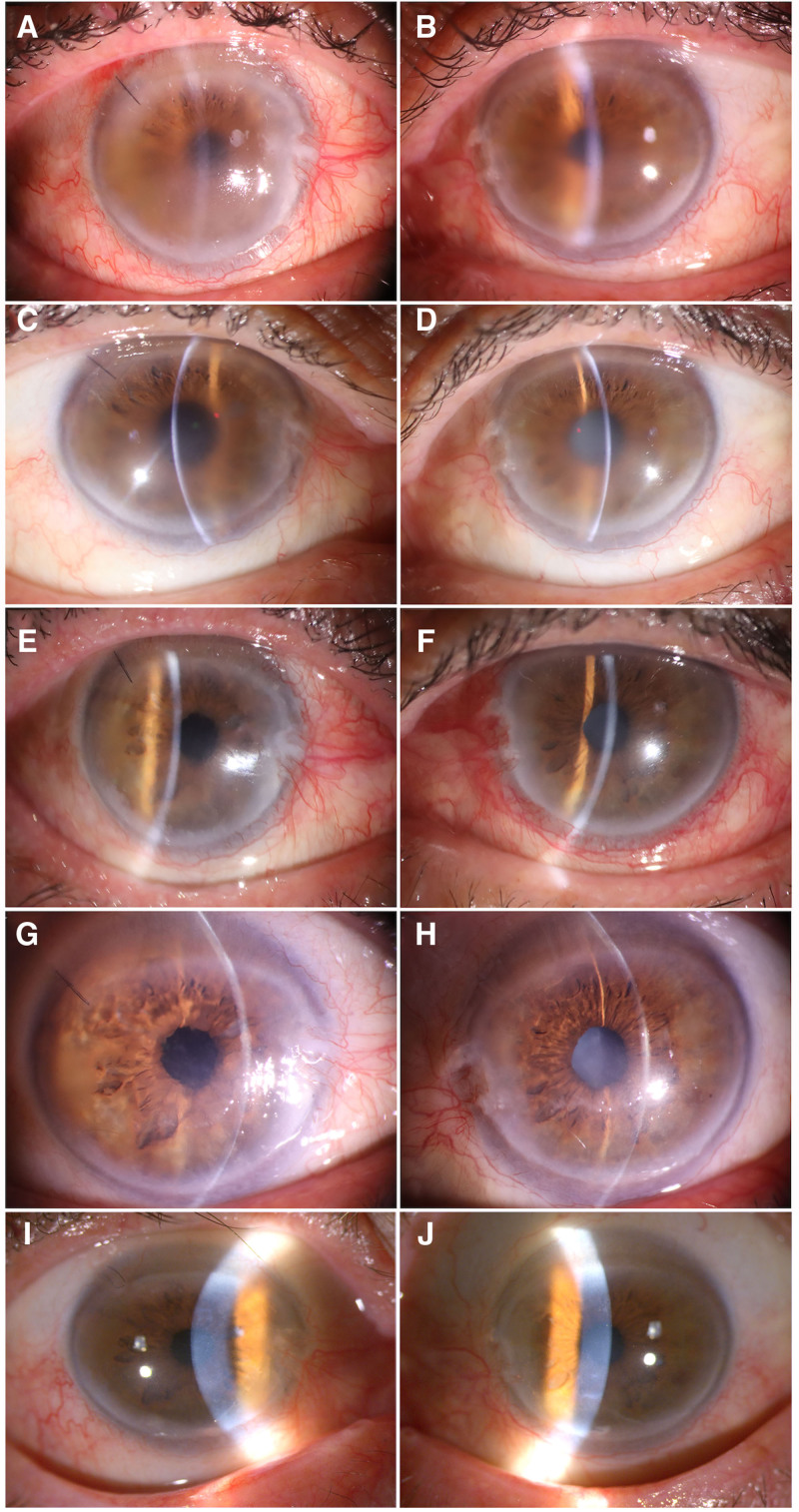
(A and B) First day after cataract surgery in the right eye with good recovery of the corneal epithelial layer; Oil droplet-like foreign body visible in the center of the cornea of the left eye. (C and D) Three months after cataract surgery in the right eye, the corneal epithelium has recovered well, no change in the left eye. (E and F) On the third postoperative day after surgery in both eye with good recovery of the corneal epithelial layer. (G and H) One week anterior elastic lamina scraping in both eye, The corneas of both eyes go clear in the center, with turbidity visible in the periphery. (I and J) Final follow-up imaging, demonstrating sustained central corneal transparency and stable peripheral opacification in both eyes.

Corneal epithelial dystrophy could not be completely ruled out at this stage, so to further the differential diagnosis with CDK, we performed epithelial scraping and cataract surgery on the right eye. Surgery is not given for the left eye for the time being because the condition is more severe in the left eye. However, after scraping the epithelium, the anterior chamber is still unclear, and the corneal epithelium is repaired quickly on the next day, which indicates that the corneal endothelial function is basic health (Fig. 4A). This again proves that the turbidity is located in the Bowman and shallow matrix layers. One week later, we scraped Bowman in the center of the pupillary area, although the corneal endothelium remained unmeasured. Fortunately, the patient’s visual acuity improved to 20/60. The right eye needed observation; the patient was advised to be discharged from the hospital to observe the recovery of the right eye and to determine the surgical approach for the left eye at an opportune time. Four weeks following the procedure, the visual acuity of the right eye had improved to 20/60, with a transparent central cornea and a cloudy periphery. The anterior chamber was clear. The pupil was round and sensitive to light reflection, while fundoscopic examination revealed a normal optic disc color with a cup-to-disc ratio of approximately 0.3. The retina was thin, exposing the choroidal texture. No significant changes in the left eye. After 3 months of stabilization in both eyes, with good, successful recovery in the right eye (a small amount of oil droplets were still visible in the central corneal area), we proceeded to perform upper elastic lamina scraping in both eyes (Fig. 4C and D). The Bowman layer and part of the cloudy superficial stromal layer were removed with a scalpel. Surprisingly, the first day after surgery, the patient’s left eye corneal epithelium had been repaired, and the cornea was clear in the center and cloudy in the periphery. Funduscopic examination revealed a reddened optic disc and retinal edema in the macular region. The visual acuity in the left eye was measured 20/200. Furthermore, the macular OCT scan revealed choroidal neovascularization in the left eye’s macular region (Fig. 2C and D). Consequently, the patient received intravitreal anti-vascular endothelial growth factor medication through injection.

**Figure 5.**
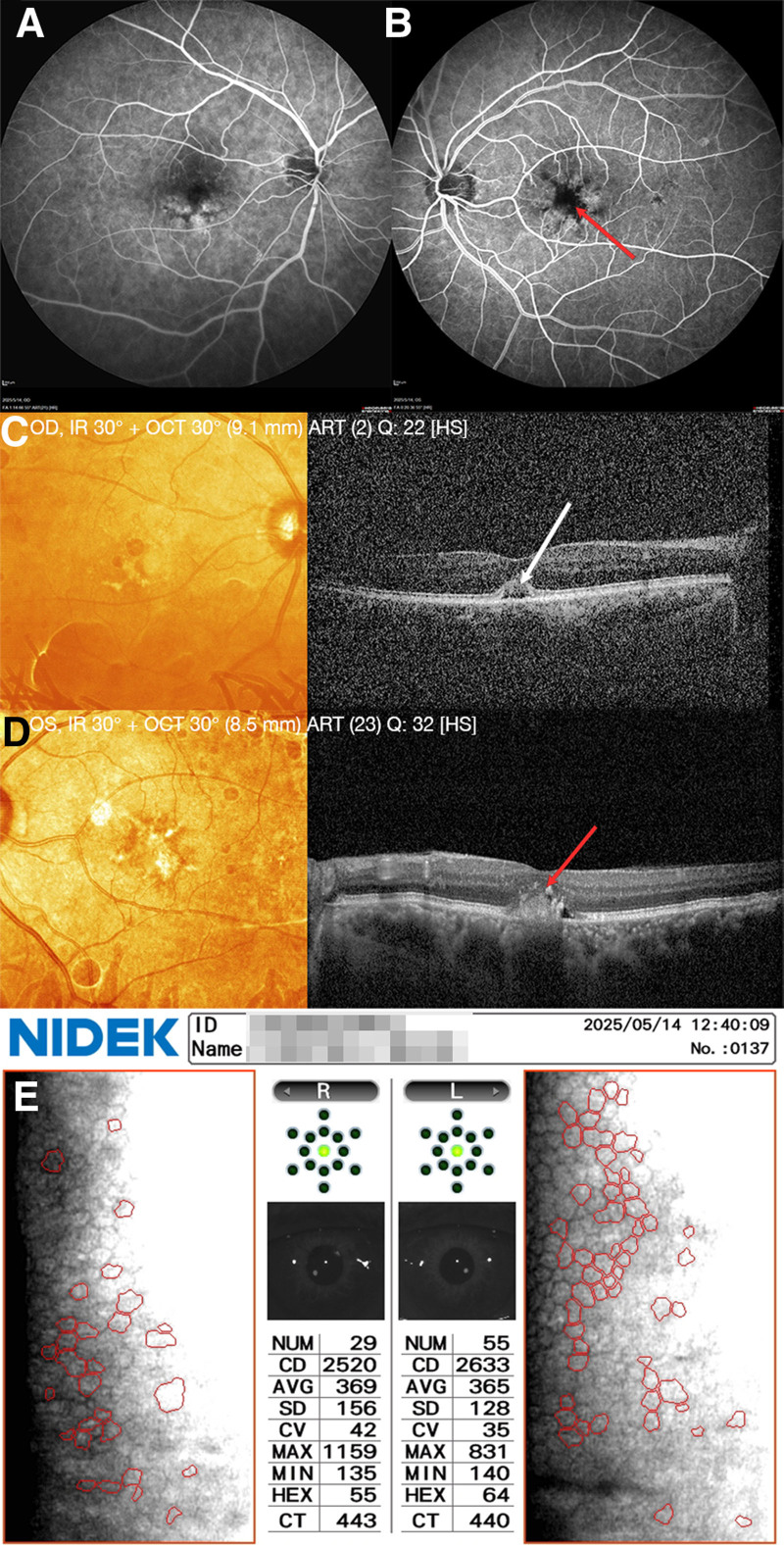
(A and B) Fluorescein angiography: Early hyperfluorescence with late-phase leakage, consistent with active choroidal neovascularization (CNV) in the left eye. (C and D) Preserved fovea with foveal pigment epithelial detachment and RPE layer drusen in the right eye (shown by white arrows). Preserved foveal center with CNV extending through the RPE layer, localized neurosensory detachment, partial ellipsoid zone disruption, and adherent epiretinal membrane (shown by red arrows). (E) Normal corneal endothelial function. RPE = retinal pigment epithelium.

The third day after surgery, a Physical examination revealed that the visual acuity in the right eye was 20/60; the left eye was 20/200. We also observed that the central cornea was clear, with precorneal opacity in both eyes (Fig. 4E and F). It had a clear anterior chamber, a round pupil, positive optical feedback, about 3 mm in diameter, positive intraocular lens position, and reddish optic disc; the retina was thin, exposing the choroidal texture, but retinal edema in the macular area and an unrevealed fovea in the left eye. The patient had improved vision in both eyes, self-care life, and no obvious eye discomfort, and the patient was discharged 3 days later. One month after discharge, both eyes were found to be stable with good corneal recovery (Fig. 4G and H); the patient was advised to undergo regular follow-up and cataract surgery on the left eye. On July 21, 2024, cataract surgery was performed on the left eye at a local hospital, though detailed postoperative outcomes were not formally documented. On May 14, 2025, the patient returned to our hospital for follow-up evaluation. Biomicroscopic slit-lamp examination revealed transparent central corneas with peripheral corneal haze in both eyes (Fig. 4I and J). Pupils were isochoric and measured 3 mm in diameter under ambient lighting, with well-centered intraocular lenses observed in the posterior chamber. The visual acuity was 20/40 in the right eye and 20/50 in the left eye. Corneal endothelial function remained intact in both eyes (Fig. 5). The macular OCT demonstrated drusen formation beneath the retinal pigment epithelium layer bilaterally (Fig. 5C and D). Notably, fluorescein angiography demonstrated hyperfluorescence with late-phase leakage in the left eye (Fig. 5A and B), consistent with active choroidal neovascularization. Based on these findings, the diagnosis of wet age-related macular degeneration in the left eye was established. Subsequently, bilateral intravitreal injections of anti-vascular endothelial growth factor agents were administered as a therapeutic intervention.

The study was approved by the Medical Ethics Committee of Xinjiang 474 Hospital. Written informed consent was obtained from the patient for publication of this case report.

## 3. Discussion

In 1973, Freedman first proposed the term “climate drop corneal degeneration (CDK)” to emphasize CDK, the leading role of external environmental factors, including ultraviolet, dryness, sand, and the characteristic change of drop deposits under the Bowman layer of the cornea^[5]^ The patient we reported was a herder living in the alpine region of Altay, Xinjiang, China. The patient was admitted to the hospital with visual blur in both eyes. The initial evidence considered corneal decompensation and cataracts, but the patient’s corneal manifestations look like corneal epithelial dystrophy. In the context of unsupported imaging evidence, we considered other measures to analyze and diagnose the opacity of the cornea. However, as a severe cataract complicated the patient’s condition, this lesion also seriously affects the patient’s visual acuity and diagnosis. Therefore, we scraped the corneal epithelium during the right eye cataract surgery. The cornea was still cloudy, but the epithelial repair speed was normal. In the second operation, we scraped off the Bowman layer and the shallow matrix layer of the right eye. The postoperative epithelium could also be repaired, and the vision could be improved. Therefore, the corneal lesions focus on the superficial matrix layer, thus excluding the disease of the corneal endothelium and the corneal epithelium.

The patient was obtained from Altay, a cold region of Xinjiang, and AS-OCT revealed that the Bowman layer reflects reflection. So, the final diagnosis is CDK in both eyes and level II. CDK was found in Kazakh individuals. After multiple regression model analyses demonstrated that age, exposure time, exposure protection, and vegetable intake were correlated with CDK.^[6]^ This disease is more binocular in outdoor workers with certain regional males.^[7]^ In this case, the patient was male, which is consistent with the characteristics reported in the literature. It is important to consider these factors to decide upon a definitive treatment option. Based on the available data, we decided to remove the lens and perform a diagnostic corneal epithelial scraping to determine the presence of postoperative corneal endothelial loss. If the damaged corneal epithelium does not recover well after surgery, the diagnosis can be clarified as corneal endothelial loss, and corneal transplantation is considered.

## 4. Conclusion

In summary, CDK is the result of a variety of synergistic factors; the specific cause is not completely clear, and its pathogenesis needs to be further clear, but most studies show that corneal epithelial injury and oxidative stress in CDK plays an important role, how to avoid corneal damage to delay or even reverse the generation of corneal deposits is an effective way to prevent and treat CDK for epidemiology, corneal morphology, sediment composition and ocular surface. There is an urgent need to identify the reason for any condition affecting the vision. If there is insufficient evidence, diagnostic surgical treatment can be conducted. Moreover, we must also consider the complications, such as corneal epithelial nonunion, corneal decompensation, and the failure to remove the lens due to the corneal opacity. When managing complex CDK requiring surgical intervention, Ophthalmologists should anticipate the need for staged therapeutic interventions, such as sequential anterior lamellar keratectomy, to achieve progressive clearance of subepithelial deposits while preserving corneal integrity, as exemplified in this case. The present case indicates that ophthalmologists must be open-minded when diagnosing and treating their patients. While seeking solutions to ophthalmological problems, ophthalmologists should also consider systemic issues rather than solely focusing on problems associated with the eyes.

Furthermore, the impact of systemic problems can extend into local areas. Consequently, physicians must consider their options from a regional and systemic point of view. The successful treatment of the current case highlights the importance of analyzing difficult ophthalmic cases by combining multidisciplinary therapy, diagnosis, different treatment options, and the relative benefits for the patient. These considerations will allow us to generate a safe and effective, feasible treatment plan.

## Acknowledgments

We acknowledge the contributions of specific colleagues, institutions, or agencies that aided the efforts of the authors.

## Author contributions

**Data curation:** Dai Kun Lei, Ying Zhou.

**Funding acquisition:** Xia Li.

**Methodology:** A bu du sa ta er Ai shan, Dai Kun Lei.

**Resources:** A bu du sa ta er Ai shan, Dai Kun Lei.

**Supervision:** Ying Zhou.

**Validation:** Xia Li.

**Writing – original draft:** A bu du sa ta er Ai shan.

**Writing – review & editing:** Xia Li.
